# Passenger lymphocyte syndrome following organ and stem cell transplantation: current insights and clinical implications

**DOI:** 10.3389/fimmu.2026.1814252

**Published:** 2026-06-11

**Authors:** Zhong-yu Kang, Xue-ya Han, Chun Liu, Wei Liu, Dai-hong Li

**Affiliations:** Department of Blood Transfusion, Tianjin First Central Hospital, School of Medicine, Nankai University, Tianjin, Nankai, China

**Keywords:** ABO incompatibility, alloimmune complication, graft-related autoimmunity, hematopoietic stem cell transplantation, passenger lymphocyte syndrome, post-transplant hemolysis, solid organ transplantation

## Abstract

**Background:**

Passenger lymphocyte syndrome (PLS) is an alloimmune complication of ABO-mismatched solid organ or hematopoietic stem cell transplantation characterized by the production of antibodies by donor-derived B lymphocytes against the recipient’s red blood cell antigens. Its incidence, risk factors, and management remain poorly defined because many cases are subclinical, and most reports are case based. This study aimed to summarize the available literature on PLS, focusing on its incidence, immunohematologic mechanisms, clinical features, and management.

**Methods:**

We systematically searched PubMed and Embase for publications up to August 2025 and included case reports, case series, and cohort studies describing PLS after kidney, liver, lung, intestinal, and hematopoietic stem cell transplantation. Data on epidemiology, ABO constellation, antibody specificity, clinical presentation, treatment, and outcomes were extracted.

**Results:**

We identified 64 studies reporting 135 cases of PLS. All were observational comprising 78% case reports/series and 22% retrospective cohort. 17 studies involved kidney transplantation, 23 liver, 7 lung, 6 intestinal, 1 heart, and 10 hematopoietic stem cell transplantations. The reported incidence ranged from approximately 9% in kidney to approximately 30–40% in liver and over 50% in lung transplantation. ABO isoagglutinins were the predominant antibodies, while Rh, Kidd, Kell, and Lewis specificities were also implicated. Most cases presented within 1–3 weeks post-transplant with abrupt hemolysis, which was usually self-limiting; however, severe cases required transfusion support, plasma exchange, or rituximab. Mortality was rare but occurred in delayed or fulminant cases.

**Conclusions:**

PLS is an under-recognized post-transplant complication with variable incidence and outcomes across organ types. Standardized diagnostic criteria, evidence-based management strategies, and multicenter studies are needed to guide clinical practice and improve patient outcomes.

## 1 Introduction

Transplantation of solid organs and hematopoietic stem cells is an established therapeutic modality for end-stage organ failure and hematologic disorders (1–3). Although advances in surgical techniques, immunosuppressive regimens, and supportive care have improved short- and long-term outcomes, transplantation is still associated with significant complications (4). Notably, hematologic complications such as anemia, thrombocytopenia, coagulopathy, and immune-mediated cytopenias are an important source of morbidity and mortality (5, 6). One such complication is passenger lymphocyte syndrome (PLS), which is a graft-versus-host–like alloimmune reaction. Although it was regarded as a subtype of graft-versus-host disease in earlier literature, its pathophysiology is distinct, as it is mediated by donor-derived B cells producing alloantibodies against recipient red blood cells and platelets. PLS often occurs after minor ABO-incompatible solid organ or bone marrow transplantation (7, 8). Beyond red cell complications, alloimmune thrombocytopenia has also been reported, driven exclusively by human platelet antigen (HPA)-1a antibodies (9, 10). PLS was first observed in the 1960s by Starzl and Marchioro during early liver transplantation studies, and was formally characterized as a distinct clinical entity by Stevens, Callender, and Jilly in 1981. It most commonly occurs in the context of minor ABO mismatch but may also involve antibodies of the Rh, Kidd, Kell, and Lewis systems (11–13). By producing clinically significant alloantibodies, donor “passenger” lymphocytes can cause abrupt hemolysis in the early post-transplant period (14–16).

PLS has been reported in various transplant settings, including kidney, liver, lung, heart, small intestine, and hematopoietic stem cell transplants (7, 17, 18). The incidence and clinical spectrum of PLS vary substantially across transplant types. It is least common after kidney transplantation, occurs more frequently after liver transplantation, and its highest incidence has been observed after combined heart–lung transplantation (18–25). PLS usually develops within 1–3 weeks post-transplant and is characterized by abrupt hemolysis (26). Most cases are self-limited, presenting with jaundice and anemia as the predominant manifestations, while a minority develop severe intravascular hemolysis that may progress to renal failure, graft dysfunction, or even death (27). Laboratory features include anemia with elevated bilirubin and lactate dehydrogenase levels, reduced haptoglobin, and a positive direct antiglobulin test (DAT) (28). Serological assays, such as antibody screening, identification, and elution studies, may reveal donor-derived antibodies against recipient red cells and provide supportive evidence for diagnosis (5, 26, 29). Although many cases of PLS resolve spontaneously as donor lymphocytes are cleared, its recognition is often difficult because hemolysis can mimic acute rejection, infection, or drug-induced cytopenia, leading to frequent underdiagnosis (30, 31). Furthermore, treatment strategies remain heterogeneous, ranging from supportive transfusion with donor-type red blood cells to plasma exchange, intensified immunosuppression, or B-cell–targeted therapy such as rituximab (8, 18, 19, 32).

Although the pathogenesis of PLS has been clarified, substantial uncertainties persist regarding its epidemiology, diagnosis, and management. Most reports are case-based or small case series, with only a few cohort studies providing systematic data, leaving currently available overall incidence estimates unreliable and organ-specific risks poorly defined. Furthermore, the presence of donor-derived blood group antibodies does not invariably translate into clinically significant hemolysis; while a considerable proportion of recipients develop detectable antibodies or DAT positivity after ABO-mismatched transplantation, only a minority progress to overt hemolysis (20, 23). This variability, likely influenced by the magnitude of antibody production, underscores the difficulty of establishing standardized diagnostic criteria for PLS. The sensitivity of DAT is limited, particularly in mild cases or when other causes of anemia coexist, and testing practices vary between centers, contributing to frequent under- or misdiagnosis (23, 24, 33). These inconsistencies underscore the need for standardized diagnostic approaches and evidence-based management.

This review aims to provide a comprehensive overview of PLS in both solid organ and hematopoietic stem cell transplantation, highlighting its epidemiology, immunohematologic basis, clinical spectrum, and management, and to outline its implications for graft function and transplant outcomes.

## 2 Methods

A structured literature search was conducted to identify published reports describing passenger lymphocyte syndrome (PLS) in the context of solid organ transplantation (SOT) or hematopoietic stem cell transplantation (HSCT). We searched PubMed (MedLINE) and Embase from database inception to August 2025. The search strategy combined controlled vocabulary and keywords related to PLS and transplantation, including: “passenger lymphocyte syndrome”, “passenger lymphocyte”, “immune hemolysis”, “alloimmune hemolysis”, “ABO mismatch”, “solid organ transplantation”, “liver transplant”, “kidney transplant”, “lung transplant”, “intestinal transplant”, and “hematopoietic stem cell transplantation”. Reference lists of relevant articles and reviews were also hand-searched to identify additional publications.

Inclusion criteria were: (1) human studies; (2) original reports describing cases of PLS occurring after kidney, liver, lung, heart, intestinal, or HSCT; and (3) articles providing clinical, serologic, or laboratory evidence supporting the diagnosis of PLS. Exclusion criteria included: non-human studies, review articles, editorials, conference abstracts without primary data, and reports lacking sufficient clinical detail to confirm PLS.

Cases were classified into two categories: (i) clinically significant hemolytic PLS, requiring a positive DAT, identification of donor-derived alloantibodies, and biochemical evidence of hemolysis; and (ii) serologic PLS, defined by a positive DAT and/or detectable donor-derived antibodies without overt hemolysis. A minor blood group incompatibility between donor and recipient was required for inclusion. Cases attributable to alternative etiologies such as acute rejection, thrombotic microangiopathy, infection, or drug-induced cytopenia were excluded unless PLS was independently confirmed through serologic evidence.

Two reviewers independently screened titles and abstracts, followed by full-text review of potentially eligible articles. Discrepancies were resolved by discussion. For each included study, we extracted data on study design, organ type, donor–recipient ABO constellation, implicated antibodies, time to onset, clinical manifestations, laboratory findings, treatment strategies, and outcomes. Because most included publications were case reports or small case series, no formal risk-of-bias or quality assessment tools were applied, consistent with prior narrative reviews of rare transplant complications (8, 16).

Included studies were stratified by organ type, and findings were synthesized descriptively, summarizing epidemiology, immunohematologic mechanisms, clinical presentation, and management across transplant settings.

## 3 Results

**Figure 1:**
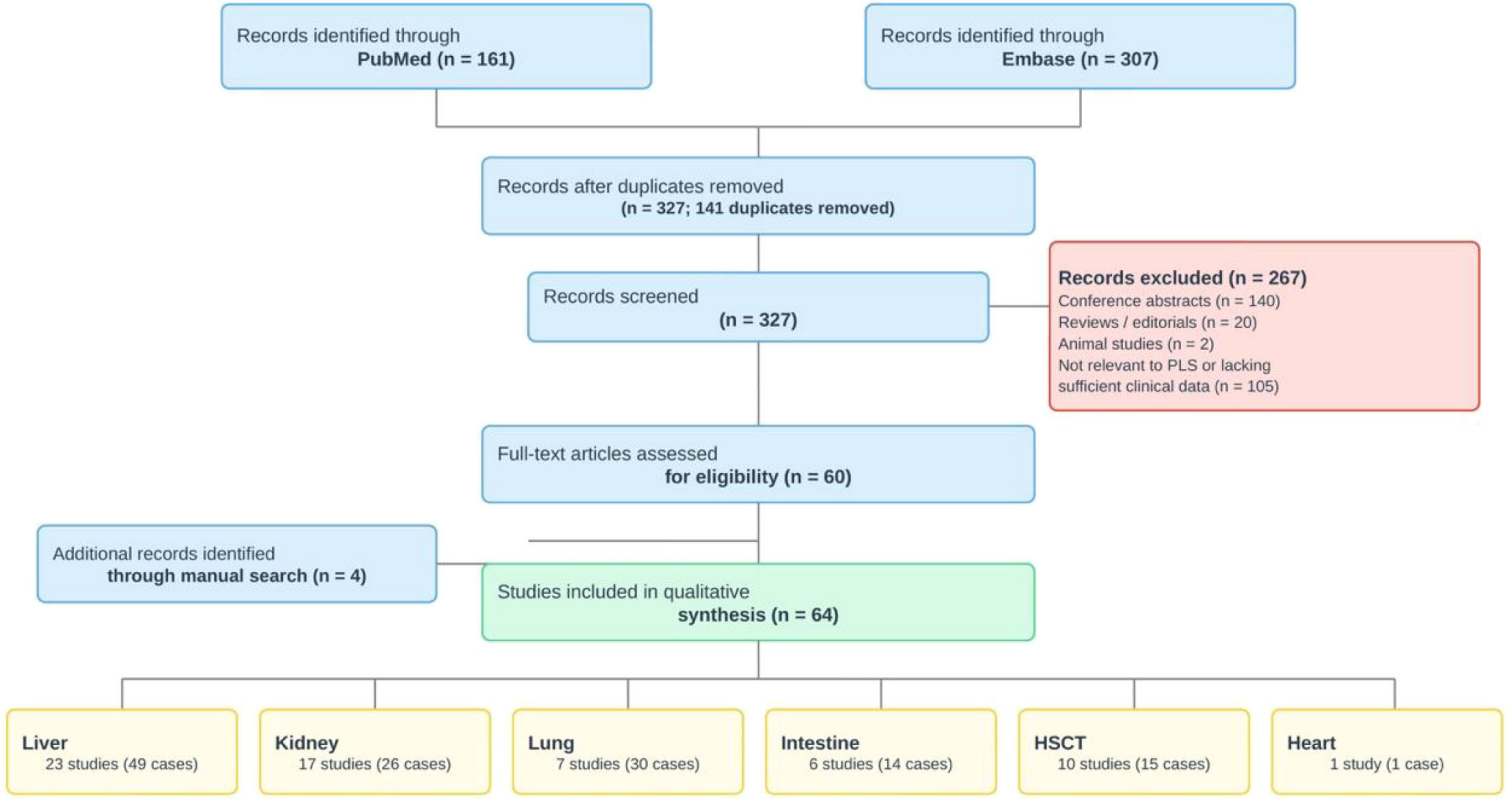
Flow diagram of study selection for the systematic review of passenger lymphocyte syndrome (PLS) in solid organ and hematopoietic stem cell transplantation.

Our systematic literature review identified 468 eligible articles published between 1977 and 2025 that specifically addressed PLS in the context of human transplantation. After removing duplicates, reviews, animal studies, and reports without original patient data, 64 studies were finally selected for detailed analysis. 19 (29.7%) of these publications were published after 2020, reflecting increasing recognition of PLS in recent years. Across the included studies, most reports were single-case descriptions or small case series, although several retrospective or prospective cohort studies were also identified. The studies showed variability in diagnostic criteria, clinical presentation, and management approaches. A total of 64 studies were included, comprising 54 solid organ transplantation (SOT) and 10 hematopoietic stem cell transplantation (HSCT) reports. The distribution by organ type is detailed in **Figure 1**. Restricting analyses to SOT, these reports encompassed 120 cases of PLS, comprising 65 males (54.2%) and 55 females (45.8%). Among solid organ transplants, PLS cases were distributed as liver (n = 49; 40.8%), kidney (n = 26; 21.7%), lung (n = 30; 25.0%), intestinal (n = 14; 11.7%), and heart (n = 1; 0.8%). Additionally, 15 cases (11.1%) occurred following HSCT. Where donor–recipient ABO constellations were reported, minor ABO incompatibility was the predominant pattern, most frequently involving group O donors transplanted into non-O recipients. Consistent with this pattern, most cases were mediated by ABO antibodies, most commonly anti-A, and anti-B. On the other hand, RhD antibodies were less frequently reported, while only sporadic cases involved other minor blood group antibodies such as anti-Jk^a^, anti-k, and anti-Fy^a^.

### 3.1 Aggregated analysis across transplant types

Although HSCT-associated PLS is immunologically distinct from SOT-associated PLS (see Section 3.5), HSCT cases were included in the global denominator for descriptive aggregation. Where relevant, SOT-specific, and HSCT-specific data are reported separately. To provide a quantitative synthesis, we aggregated key clinical parameters from all 135 reported cases (120 SOT and 15 HSCT) (**Tables 1**, **2**). Regarding antibody specificity, ABO antibodies (anti-A, anti-A_1_, anti-A, B, and anti-B) were the most common, accounting for 73.5% of liver, 88.4% of kidney, approximately 84% of lung, 100% of intestinal, and 80% of HSCT cases. Across all transplant types, ABO antibodies were implicated in approximately 82.2% (110/135) of cases. Rh antibodies (predominantly anti-D, with occasional anti-C and anti-E) accounted for 14.3% (19/135) of cases overall, with the highest proportion in liver transplantation (22.5%). Other antibody specificities, including anti-Jk^a^, anti-K, anti-Fy^a^, anti-HPA-1a, anti-HPA-3a, and donor-derived HLA antibodies, were reported in approximately 6.7% of cases (10, 25, 37, 38, 43, 56).

**Table 1:** Characteristics of PLS in diverse organ transplantations.

Transplant type	Incidence/reported cases	Main antibodies	Typical onset	Clinical manifestations	Treatment and prognosis
Liver transplantation	2.8% overall to 40% among minor ABO-incompatible recipients; wide variation reflecting differences in study design and sample size; 49 cases across 23 studies.	Predominantly anti-A and anti-B; less frequent anti-D, anti-K, anti-Fy^a^, anti-Jk^a^, or HPA antibodies.	Usually 1–3 weeks post-transplant; delayed cases reported.	Hemolytic anemia with positive DAT, often accompanied by jaundice; occasional thrombocytopenia.	Usually self-limiting; donor-type RBC transfusion is common, with plasmapheresis used in severe cases.
Kidney transplantation	Approximately 9% in minor ABO-mismatched transplants; 26 cases across 17 studies.	Mostly anti-A and anti-B; occasional anti-D, E or anti-Jk^a^.	Usually 5–15 days post-transplant.	Hemolytic anemia with positive DAT; occasional GI ischemia or Coombs’-negative hemolysis.	Usually self-limiting; most require transfusion, steroids or plasmapheresis in severe cases.
Lung transplantation	5–31% in minor ABO mismatch (varies by recipient blood type); 30 cases across 7 studies.	Mostly anti-A (including anti-A1); less common anti-B, anti-D, anti-C; rare anti-HLA and anti-Kell.	Usually 1–2 weeks; delayed cases up to 120 days.	Hemolytic anemia with jaundice and positive DAT; occasionally complicated by thrombotic microangiopathy.	Responsive to donor-type RBC transfusion; plasmapheresis for severe cases; transfusion needs may be high.
Intestinal transplantation	Rare but high risk due to abundant lymphoid tissue; 14 cases across 6 studies.	Mainly anti-A and anti-B.	Within 1–2 weeks post-transplant.	Severe hemolytic anemia with positive DAT, often with jaundice.	Often requires aggressive transfusion support and plasmapheresis; higher severity in a subset.
Hematopoietic stem cell transplantation	Reported in ~5–15% of minor ABO mismatches; 15 cases across 10 studies.	ABO antibodies (anti-A, anti-B) most common; occasional anti-D, anti-Jk^a^.	Usually within 1–3 weeks post-transplant; can be delayed for months.	Hemolytic anemia with jaundice and positive DAT; sometimes prolonged or recurrent.	Usually self-limiting; repeated transfusions or immunotherapy may be needed for prolonged cases.
Heart transplantation	1 case	ABO antibodies (anti-A)	Within 1–3 weeks post-transplant	Hemolytic anemia with positive DAT	Responsive to donor-type RBC transfusion; generally self-limiting

DAT, direct antiglobulin test; HSCT, hematopoietic stem cell transplantation; GI, gastrointestinal; GVHD, graft-versus-host disease.

**Table 2:** Aggregated summary of PLS characteristics across transplant types.

Parameter	Liver (n=49)	Kidney (n=26)	Lung (n=30)	Intestinal (n=14)	HSCT (n=15)	Heart (n=1)	Overall (n=135)
ABO antibodies	73.5%	88.4%	~84%	100%	80%	100%	82.2%
Rh antibodies	14.3%	11.5%	9.7%	0%	13.3%	0%	11.1%
Other antibodies	12.2%	0%	6.5%	0%	6.7%	0%	6.7%
Median onset	7–14 days	17 days	7–21 days	4–15 days	7–21 days	18 days	1–3 weeks
Transfusion required	Majority	Majority	Majority	Majority	Majority	Yes	>70%
Immunomodulatory Tx	~25%	~25%	Rare	~30%	~20%	No	~25–30%
Graft loss	0	1	0	0	0	0	1 (<1%)
Deaths	0	0	2	2	1	0	5 (3.7%)

Percentages are calculated using organ-specific or total case numbers as denominators. Not all variables were uniformly reported; estimates for transfusion requirement and immunomodulatory therapy are based on cases with available data.

The time to PLS onset was consistently within 1-3 weeks post-transplant across all organ types. In kidney transplantation, the median onset was 17 days (range, POD 5 to 3 months) (48). The earliest onset was reported in intestinal transplantation (POD 4) (67), whereas the latest documented onset occurred after lung transplantation (120 days) (22).

The majority of cases required RBC transfusion support. Immunomodulatory therapy beyond supportive transfusion - including corticosteroids, plasmapheresis, IVIG, or rituximab - was employed in approximately 25-30% of cases, predominantly in severe or refractory presentations. Graft loss directly attributable to PLS was documented in only one kidney transplant recipient, in whom refractory PLS was complicated by antibody-mediated rejection (79). Five deaths were identified across all 135 cases (3.7%): two after lung transplantation, two after intestinal transplantation, and one after HSCT. Notably, in most instances, death was primarily attributable to concurrent complications (infection, multiorgan failure) rather than hemolysis alone, and direct PLS-attributable mortality thus appears to be very low.

All percentages in the aggregated analysis were calculated based on the total number of cases per organ type or overall (n/N), regardless of data availability. Given the predominance of case reports, not all variables were uniformly reported across studies. Antibody specificity was available in 135/135 cases (100%), time to onset in 127/135 (94.1%), transfusion requirement was available in most cases but was heterogeneously reported, and mortality data were available in 135/135 cases (100%). Immunomodulatory therapy use could not be precisely quantified due to heterogeneous reporting, and the estimate of 25–30% should be interpreted as approximate.

### 3.2 Liver transplantation

Our review identified 23 studies encompassing 49 liver transplant recipients with PLS (**Table 3**). Most cases were secondary to ABO antibodies 73.5% (36/49), followed by Rh antibodies 4.3% (7/49) and other minor blood group antibodies, including Kidd, Kell, Duffy, Lewis, and HPA 12.2% (6/49). Nearly all patients presented with clinically significant hemolysis, while only a few represented non-hemolytic variants. The reported prevalence of PLS in liver transplantation showed significant variability. De Bruijn et al. observed hemolysis in 40% of minor ABO-incompatible recipients (4/10), while ElAnsary et al. reported 18% in a prospective series (2/11) (32, 34). In a large Brazilian cohort, Brunetta et al. found PLS in 2.8% of 175 recipients, with minor ABO mismatch being the major risk factor (35). In adults, Romero et al. described 12 cases among 1217 transplant recipients (17.9% of minor ABO-incompatible and 1.4% of Rh-incompatible transplants) (18). In pediatric populations, Woolfson et al. identified PLS in 14% of ABO-compatible grafts (7/51), all in A+ recipients with O+ donors (36). The higher incidence of PLS in liver transplantation compared with other solid organs has been attributed to the large mass of transferred lymphoid tissue, the presence of donor-derived B-cell activating factor (BAFF) which supports B-cell survival and antibody production, the high frequency of non–ABO-compatible grafts, and the massive transfusions often required during treatment (5, 15, 18, 35).

**Table 3:** Characteristics of passenger lymphocyte syndrome after liver transplantation.

Study (year)	Cases (n)	Recipient type	Donor type	Antibody	DAT	Treatment	Outcome	Transfusion
Brunetta et al. (2017) (19)	2	A+/B+	O+	anti-A1, anti-B	Yes	Case1: 4U RBC (2×A2, 2×O) + supportive; Case2: folate + hydration; no RBC	Both stabilized and discharged; Hb 11.1 g/dL by Day 26 in case 2	Yes
Brunetta et al. (2020) (35)	5	A/B	O	anti-A1, anti-B	Yes	Supportive; donor-type RBC as needed (3/5 PLS transfused)	Good overall; immune hemolysis rate low	Yes/No
Chase et al. (2024) (38)	1	HPA-1a/1a	HPA-1b/1b	anti-HPA-1a	NR	57U platelets; splenectomy; rituximab; plasma exchange; IVIG; eltrombopag; romiplostim; efgartigimod	Platelet count recovery with efgartigimod synergy; clinical stabilization	Yes
de Bruijn et al. (2017) (32)	4	AB-/A+/B+	O+	anti-A1, anti-B	Yes	Severity-guided: donor-type RBC; supportive; variable IS adjustments	All 4 resolved; no PLS-related mortality reported	Yes
ElAnsary et al. (2015) (34)	2	B+/D+	O+/D-	anti-B, anti-D	Yes	Supportive; donor-type RBC as needed; monitoring DAT/eluate	PLS generally self-limited within early post-LT window; no PLS-attributed mortality reported	Yes
Sönnerborg et al. (2017) (9)	1	O+	O+ HPA-1b	anti-HPA-1a	NR	HPA-1a negative platelets; IVIG; rituximab; thrombopoietin	Recovered, antibodies cleared	Yes
Lindholm et al. (2018) (10)	1	A+	A+, HPA-1b/1b, anti-HPA-1a+	anti-HPA-1a	NR	Platelet transfusion, IVIG, steroids	Recovered, platelet normalized POD28	Yes
French et al. (2020) (37)	1	HPA-3a/3a	HPA-3b/3b	anti-HPA-3a	NR	IVIG 1 g/kg (Day +9) with rapid, sustained PLT recovery; supportive care	Stabilized; recovery of PLT count; no donor chimerism detected (Days +1 & +35)	Yes
Gniadek et al. (2017) (70)	1	O+	O-	anti-D	Yes	Switch to D− RBCs; IVIG/steroids as per course; supportive	Anemia resolved; DAT persisted to POD233; no alternative cause found	Yes
Hareuveni et al. (2002) (71)	1	O+Jk(a+)	O-Jk(a−)	anti-Jk^a^	Yes	Switched to Jk(a−) RBCs; supportive; immunosuppression ongoing	Hemolysis subsided; DAT weak+ Day 35; serum Ab negative at 6 months; DAT weak+ persists	Yes
Jacobs et al. (2021) (40)	1	AB+	B+	anti-A	Yes	Switch to type O RBCs; intensified immunosuppression; supportive; systems fix (notify all ABO mismatches)	Stabilized and discharged; biomarkers normalized after switch	Yes
Peck et al. (2015) (72)	1	AB+	B+	anti-A1	Yes	Prednisone 40 mg bid; switch transfusion to O+ PRBCs; supportive care	No further hemolysis after O+; stable discharge POD13; Hb normalized by 9 months	Yes
Jung et al. (2018) (41)	1	A1B +	B+	anti-A	Yes	Transfuse 4U O RhD+ RBCs; supportive; later plasmapheresis ×6 for AMR/biliary	Clinical labs improved; A1 phenotype recovered day 61; discharged	Yes
Mathavan et al. (2024) (33)	1	A+	O+	anti-A	Yes	Supportive; donor-matched O RhD+ transfusions; continued IS	Stabilized; Hb 10.6 g/dL at 1 mo; chimerism negative for donor cells	Yes
Monfort et al. (2015) (39)	1	A-	O+	anti-A	Yes	Switched to group O, D-C-E-c+e+ K− Le(a−) RBCs; high-dose steroids; anti-CMV IVIG	Improved; transfusion-independent by ~day 40; Hb 14.7 g/dL at 3 months	Yes
Nguyen et al. (2022) (73)	1	AB+	AB-	anti-D	Yes	Supportive care; donor-compatible RBC as needed; prednisone	Stabilized; no PLS-attributed mortality reported	Yes
Romero et al. (2015) (18)	12	A/B/O/AB	A-/B+/O-/O+	anti-A, anti-B, anti-D	Yes	Increase corticosteroids; donor-compatible RBC transfusion (median 4U)	Good overall; no PLS-related deaths reported	Yes
Sachan et al. (2018) (74)	1	AB+	O+	anti-A	Yes	Transfused 6U O+ PRBC between POD10 and 20; supportive; IS per protocol	Gradual improvement; discharged in stable condition	Yes
Sandler et al. (2017) (42)	1	O D− C−	O D− C−	anti-D, anti-C	NR	NR	NR	NR
Seltsam et al. (2001) (43)	1	O+ K− Fy(a+)	O+ K− Fy(a-)	anti-K in serum; anti-Fy^a^ in eluate	Yes	Prednisolone, tacrolimus, basiliximab); multiple perioperative transfusions; no PLS-specific treatment (no hemolysis)	Stable	Yes
Turiño-Luque et al. (2012) (75)	1	A+	A-	anti-D	NR	Corticosteroids; transfuse RhD-compatible RBCs (A−)	Stabilized; later acute rejection grade II treated; discharged ~2 months	Yes
Woolfson et al. (2019) (36)	7	A+	O+	anti-A	Yes	Donor-group PRBC (median 2U); no escalations required	All recovered; no deaths; some needed second transfusion within 3–4 days	Yes
Zhao et al. (2024) (27)	1	A+	O+	anti-A	Yes	Cross-matched O+ washed RBC (4U) + IS optimization; supportive	Stabilized; symptoms resolved; maintained Hb ~70 g/L during course	Yes

Most cases involve anti-A and anti-B isohemagglutinins, while cases involving anti-D and other Rh antibodies are well documented. Several reports have described non-ABO/Rh alloantibodies such as anti-Jk^a^, anti-K, and anti-Fy^a^, and platelet-specific antibodies including anti-HPA-1a and -HPA-3a (18, 37–39). Disease onset is usually 1–3 weeks post-transplantation. Most patients developed abrupt anemia with Hb nadirs around 6–8 g/dL, often requiring transfusion. Jaundice and indirect hyperbilirubinemia were common (27). Severe cases progressed to acute kidney injury, cardiovascular collapse, or multiorgan failure (40). Rare variants included immune thrombocytopenia, transient loss of A1 phenotype, and non-hemolytic PLS where antibodies are detected without anemia (37, 41, 42). Diagnosis relies on evidence of hemolysis with a positive DAT and identification of donor-derived antibodies in eluates (18). Antibodies usually appear within 7–14 days after transplantation and disappear within 3 months. Subclinical cases with isolated DAT positivity are frequent (35). Molecular techniques, including short tandem repeat chimerism and PCR for donor-specific HLA alleles, have confirmed the persistence of donor lymphocytes and linked them to antibody production (33, 43). Refractory thrombocytopenic variants have been successfully treated with efgartigimod (40). Pediatric cases generally followed a milder course and were controlled with supportive transfusion (36).

### 3.3 Kidney transplantation

Our review identified 17 studies encompassing 26 kidney transplantation recipients with PLS (**Table 4**). Most cases were secondary to ABO antibodies (88.4%) followed by Rh antibodies (11.5%). The incidence of PLS was approximately 9% in ABO-mismatched renal transplants (29, 44). A systematic review by Zhao et al. identified 91 published cases of PLS after renal transplantation, highlighting that although uncommon, the syndrome is clinically significant (45). Risk factors of PLS include O-to-A or O-to-B donor-recipient combinations, prior donor sensitization through pregnancy or transfusion, and cyclosporine-based immunosuppression, which appears more permissive for B-cell activation compared with tacrolimus (15).

**Table 4:** Characteristics of passenger lymphocyte syndrome after kidney transplantation.

Study (year)	Cases (n)	Recipient type	Donor type	Antibody	DAT	Treatment	Outcome	Transfusion
Achkar et al. (2011) (20)	5	NR	NR	anti-A, anti-B	Yes	Transfusion + immunosuppressive support	Required hospitalization; treated with immunosuppression and transfusion	Yes
Hedley et al. (2008) (76)	1	A+	O+	anti-A	Yes	Stop sirolimus; steroids ↑; transfuse donor-compatible; add MMF	Resolution; transfusion-independent from Day 19	Yes
Ainsworth et al. (2009) (77)	1	A+	A-	anti-D	Yes	Rh-compatible RBCs; IVIG intermittently	Needed 23U RBC over 6 months; stabilized	Yes
Debska-Slizien et al. (2003) (78)	3	A+/B+	O+/A+	anti-A anti-B	Yes	Switch CsA→Tac; decreased MMF; EPO↑; 5U RBC each (acute)	Hemolysis resolved; graft preserved	Yes
Dirim et al. (2023) (79)	1	A+	O+	anti-A1	Yes	Steroid pulses + IVIG + rituximab → refractory; immunoadsorption ×6	PLS controlled; later AMR → graft loss by ~day 60	Yes
Dubey et al. (2014) (28)	1	B+	O+	anti-B	Yes	Methylpred pulses; plasmapheresis ×2; switch to O-group PRBCs	Improved; discharge day 29 with Cr 1.2 mg/dL, Hb 8 g/dL; anti-B titer 2	Yes
ElAnsary et al. (2015) (34)	2	AB+/B+	A+/O+	anti-B	Yes	NR	No major morbidity; one RT rejection by Day +90	NR
Hurtarte-Sandoval et al. (2015) (50)	1	A+	A-	anti-D anti-E	Yes	Prednisone 1 mg/kg/d; no transfusion	Hb 7.5→9.9 g/dL in 8 days; normalized	No
Pascual-Perez et al. (2013) (80)	2	A+	O+	anti-A	Yes	4-12U PRBC + methylprednisolone 1 mg/kg/d	Stabilized; case2 had acute rejection II-B on Day 31 (thymoglobulin)	Yes
Kato et al. (2009) (81)	1	AB+	B+	anti-A	NR	Solu-Medrol pulse; washed RBC; prednisone; add MMF	Recovered; discharge POD53	Yes
Mandowara et al. (2021) (82)	1	A+	O+	anti-A	Yes	Transfuse 2U donor-group O+ PRBC; increase prednisolone (15→30 mg/d)	Hemoglobin stabilized; renal function good on follow-up	Yes
Nadarajah et al. (2013) (29)	2	A+	O+	anti-A anti-A1	Yes	Transfusion support (3U in case 1, 7U in case 2); steroids ↑; ATG used for rejection in case2	Both recovered; good graft function at discharge	Yes
Nishide et al. (2019) (83)	1	B+	A+	anti-B	NR	Steroid pulse; increase MMF to 2 g/d; convert cyclosporine→tacrolimus; transfuse 8U PRBC	PLS resolved by ~POD60; graft function maintained	Yes
Karanth et al. (2014) (47)	1	B+	O-	anti-D	Yes	Transfuse O RhD− PRBC (2U each on POD16 & POD18); no other specific therapy	Hemolysis abated; Hb normalized by 3 months; excellent graft function	Yes
Prethika et al. (2020) (68)	1	B+	A+	anti-A	Yes	O-group leukodepleted PRBC; standard IS (Tac/MMF/steroid; basiliximab); peri-Tx TPE/IVIG/rituximab	Hb and graft function improved; uneventful follow-up	Yes
Yeom et al. (2023) (49)	1	A+	O+	anti-A	Yes	Switch transfusion to donor-type O RBC; ganciclovir; ↑IS; TPE ×5; segmental colectomy + ileostomy	Hemolysis controlled; GI symptoms resolved; long-term doing well	Yes
Zhou et al. (2023) (69)	1	A+	O+	anti-A anti-B	Yes	Donor-type O RBC transfusion (total 8U); supportive care	Stabilized by POD18; uneventful graft function	Yes

Symptoms typically occur within 1–3 weeks post-transplant, with a median onset of 17 days, however, the onset of PLS could range from POD 5 to as late as 3 months (46). The hallmark presentation is abrupt anemia, often accompanied by jaundice, reticulocytosis, elevated LDH, and a positive DAT. While many episodes are mild and self-limited, severe hemolysis requiring massive transfusion support has been reported (47). Atypical features rarely occur and may include Coombs-negative autoimmune hemolysis or gastrointestinal ischemia with disseminated intravascular coagulation in pediatric recipients (48, 49). Most cases are due to donor-derived ABO antibodies, particularly anti-A in O→A transplants, reflecting the higher antigen density on A red cells (29). Anti-B antibodies are less frequent, while alloantibodies against Rh (notably anti-D and anti-E) have also been implicated (50). A previous case report illustrated that anti-D hemolysis in kidney transplant can be particularly severe, requiring intensive transfusion support (47). Nevertheless, prolonged or refractory cases have been described. Some patients maintain stable graft function despite significant anemia, while others experience graft dysfunction or loss in association with severe or resistant hemolysis (48, 49). Prophylactic administration of rituximab before ABO-mismatched kidney transplantation eliminated PLS in a large cohort, although breakthrough cases despite B-cell depletion have been observed. In extreme situations, graft nephrectomy may be required, with resolution of hemolysis after organ removal (44).

### 3.4 Lung transplantation>

Our review identified seven studies encompassing 30 lung transplant recipients with PLS (**Table 5**). The incidence of PLS after lung transplantation varied significantly across studies. According to a study by Kohl et al., PLS affected 18.18% of A-type recipients receiving O-type grafts in a retrospective cohort and 30.77% in a prospective cohort. For B-type recipients of grafts from type-O donors, the incidence was 5.13%, while 20% of AB-type recipients receiving grafts from O-type donors developed PLS. These findings underscore the high incidence of PLS in ABO minor incompatible lung transplants, particularly for A-type recipients receiving O-type grafts (22). The high incidence is largely due to the high lymphoid content of lung grafts, which contributes to the transfer of donor-derived B lymphocytes that produce antibodies against the recipient’s red blood cell antigens (51, 52). The majority of PLS cases after lung transplantation are due to ABO incompatibility, with anti-A antibodies being the most common, particularly in group A recipients receiving O grafts. Importantly, Kohl et al. demonstrated through extended immunohematologic workup including red blood cell eluate analysis that the causative antibody specificities encompassed the full ABO spectrum. Anti-A_1_ was the most frequently detected antibody by plasma IAT (81.25% of retrospective cases), while eluate testing revealed anti-A and the rare anti-A, B specificity, which recognizes an epitope common to both A and B carbohydrates, in several cases. Less commonly, anti-B antibodies have been detected. In rare cases, Rh antibodies, including anti-D and anti-C, can contribute to PLS. Furthermore, anti-K antibodies from the Kell blood group system have also been reported in some cases (51). Additionally, Fujimoto et al. highlighted the role of HLA antibodies in certain cases, suggesting that immune responses to donor HLA antigens may also play a role in the development of PLS (53). The immune response varies, and antibodies may persist in the recipient for months after transplantation.

**Table 5:** Characteristics of passenger lymphocyte syndrome after lung transplantation.

Study (year)	Cases (n)	Recipient type	Donor type	Antibody	DAT	Treatment	Outcome	Transfusion
Cserti-Gazdewich et al. (2009) (21)	3	A+/O+	A-/O-	anti-D anti-C	Yes	D− antigen-compatible RBC; plasmapheresis (POD36–40)	Case 1: Death at POD330 (indirectly related); others stable without major complications	Yes
Fujimoto et al. (2022) (53)	2	NR	NR	NR	NR	Observation under standard immunosuppression	Alive; persistent donor-derived HLA Ab without complications	Yes
Horlait et al. (2013) (51)	1	A+	O+	anti-A anti-K	Yes	Switch to O+ RBC; supportive care; immunosuppression adjusted	Resolved hemolysis; normal renal function at discharge	Yes
Kohl et al. (2024) (22)	16	A, B, AB	O+	Anti-A_1_, anti-A, anti-A, B, anti-B	Yes	Antigen-negative RBC support per protocol; monitoring	Comparable length of stay/complications; anemia more pronounced in PLS	Yes
Aujayeb et al. (2014) (52)	6	A+/B+/A-	O +	Anti-A Anti-B	Yes	NR	Case 2: Death	Yes
Low et al. (2012) (54)	1	A+	O+	anti-A1	Yes	O+ RBC support; observation	Resolved hemolysis	Yes
Sokol et al. (2002) (26)	1	A+	O	anti-A	Yes	Group O RBC transfusion; steroid escalation	Resolved over ~4 weeks	Yes

The onset of hemolysis in PLS is variable, typically occurring between 1 and 3 weeks after transplantation; however, it can also be observed up to 120 days post-transplant (21, 22, 52). Common signs include a decrease in hemoglobin levels, elevated LDH, increased bilirubin levels, and decreased haptoglobin. In severe cases, patients may experience transfusion-dependent anemia. Other clinical findings may include the presence of nucleated red blood cells, polychromasia, and spherocytosis in the peripheral blood smear (51). Diagnosis is confirmed through a positive DAT and the detection of donor-derived antibodies in the recipient’s plasma or red cell eluates (21). Immunosuppressive therapy may also be adjusted in cases of refractory hemolysis (51, 54).

### 3.5 Hematopoietic stem cell transplantation

Our review identified 10 studies comprising 15 HSCT recipients who developed PLS (**Table 6**). The publications were individual case reports, with the exception of a single cohort study by Zaimoku et al., which reported a PLS incidence of 28% (5 of 18 cases) in minor or bidirectional ABO-incompatible HSCT (55). Importantly, an earlier series by Worel et al. reported that 10–15% of minor ABO-mismatched transplants develop clinically significant immune hemolysis (56). Although not all such hemolytic events strictly fulfill the diagnostic criteria for PLS, these observations suggest that donor-derived alloantibody–mediated hemolysis is relatively frequent in this setting and that the true incidence of PLS may be underestimated.

**Table 6:** Characteristics of passenger lymphocyte syndrome after intestine transplantation.

Study (year)	Cases (n)	Recipient type	Donor type	Antibody	DAT	Treatment	Outcome	Transfusion
Obeidalla et al. (2022) (67)	1	B+	O-	anti-B	Yes	Donor-type O RBC transfusions; supportive	Self-limited; improved by 2 weeks	Yes
Thomas et al. (2024) (64)	9	NR	NR	anti-A anti-B	NR	Supportive; IS escalation is not usually needed	Self-limited; all survived	Yes
Zarei et al. (2022) (24)	1	AB+	O+	anti-A and/or anti-B	Yes	Donor-type O+ RBC; IVIG; 7 sessions plasmapheresis; IS maintenance	Hemolysis controlled; later death from catheter sepsis	Yes
Davis et al. (2011) (65)	1	A+	O+	anti-A	Yes	Donor-type O RBC; plasmapheresis ×6; monoclonal antibody therapy (alemtuzumab for liver GVHD)	Hemolysis controlled; later died from infectious complications	Yes
Foell et al. (2017) (23)	1	A+	O+	anti-A	Yes	Donor-specific O RBC; high-dose steroids; plasmapheresis ×2; later bleeding from aneurysm coiled	Resolved hemolysis	Yes
Panaro et al. (2004) (66)	1	A	O	anti-A, B	Yes	O-negative RBC (10 U/5 days); double-volume plasmapheresis ×2; rituximab 375 μg/m² ×1	Rapid improvement; no recurrence	Yes

Among the reported HSCT cases, most were attributable to ABO antibodies, with anti-A being the most frequent (57). Rh antibodies, predominantly anti-D, were the second most common, while occasional cases involved other blood group systems such as Kidd (anti-Jk^a^) (25, 58, 59). PLS after HSCT usually emerges within the first 1–3 weeks following transplantation, most often coinciding with hematopoietic engraftment (60). The syndrome is characterized by abrupt-onset hemolysis, manifested by a rapid decline in hemoglobin levels, elevated lactate dehydrogenase and indirect bilirubin, and undetectable haptoglobin (61). While many episodes are self-limited, severe cases may progress to acute renal failure, multiorgan dysfunction, or even death (62). Recurrent episodes have also been documented in patients undergoing multiple ABO-incompatible HSCTs. Furthermore, PLS has been linked to an increased risk of acute GVHD and inferior survival outcomes in prospective cohorts (55). In severe or refractory cases, rituximab has been successfully used to suppress donor-derived B cells (63). Other reported interventions include plasma exchange, exchange transfusion, and modification of calcineurin inhibitor regimens (61). The absence of methotrexate in GVHD prophylaxis has been associated with increased risk of PLS, likely due to preserved B-cell activity (62). Importantly, PLS in the HSCT setting is immunologically distinct from PLS after solid organ transplantation and warrants separate consideration. In solid organ transplantation, donor lymphocytes are finite passengers that are gradually eliminated, resulting in a self-limited course. In contrast, following HSCT, donor hematopoiesis replaces recipient immunity, and donor-derived B cells may persist indefinitely as part of immune reconstitution. This can result in prolonged or recurrent antibody production rather than the transient hemolysis typical of solid organ PLS. Additionally, ABO blood group conversion occurs as donor hematopoiesis engrafts, and hemolysis may reflect transitional immune dynamics during the period when recipient-type RBCs coexist with donor-derived antibodies. The interplay between PLS and graft-versus-host disease further complicates the clinical picture, as both processes involve donor-derived immune activation against recipient antigens. These fundamental differences in pathophysiology, duration, and clinical context underscore the importance of analyzing HSCT-associated PLS separately from solid organ PLS.

### 3.6 Intestinal transplantation

PLS is rarely reported after intestinal transplantation, despite the large volume of lymphoid tissue contained within the graft. Our review identified five case reports and one single-center series describing PLS in this setting (**Table 7**). The largest cohort, from Thomas et al., reviewed 103 intestinal transplants (31 with minor ABO mismatch) and documented nine cases of PLS (29%), all attributable to ABO antibodies, and none were fatal (64). The remainder of the literature consisted of isolated case reports, underscoring the scarcity of systematically reported data in this population. To date, the study by Thomas et al. remains the only report providing an incidence estimate, whereas all other publications describe single cases without quantifying overall frequency. Most intestinal transplant-associated PLS cases are mediated by ABO antibodies, particularly anti-A or anti-B when grafts from group O donors were transplanted into group A or B recipients (23, 24, 65, 66). However, there has been no confirmed case of intestinal PLS primarily mediated by non-ABO antibodies.

**Table 7:** Characteristics of passenger lymphocyte syndrome after HSCT transplantation.

Study (year)	Cases (n)	Recipient type	Donor type	Antibody	DAT	Treatment	Outcome	Transfusion
Adams et al. (2010) (59)	1	O+	A-	anti-D	Yes	Supportive; transfuse O RhD− RBC (12U Day +8 to +15); hydration	Self-limited; no further RBC after +15; engraftment: ANC +10, PLT +12; type A RhD− by Day +60	Yes
Hoegler et al. (2002) (84)	1	A+	O+	anti-A	NR	High-dose steroids; O RhD+RBCs (total 7U); supportive	Transfusion-independent by Day +25; anti-A declined but present to Day +90	Yes
Iwanaga et al. (2012) (85)	1	A+	O+	anti-A	No	Prednisolone; O RBC; supportive; IVIG for infection earlier	Fever resolved by Day +18; anti-A disappeared by Day +41	Yes
Lee et al. (2008) (63)	1	A+	O+	anti-A	Yes	O+ PRBCs; methylpred 2.5 mg/kg/d; rituximab 375 mg/m² Day +8	Hemolysis aborted within ~3 days; total PRBC 7U; DAT negative Day 20	Yes
Leo et al. (2000) (25)	1	Jk(a+)	Jk(a-)	anti-Jk^a^	Yes	Switch to Jk(a−) RBC; supportive transfusion	Self-limited; stable Hb after Day +24; engraftment successful	Yes
Noubouossie et al. (2022) (57)	1	A+	O+	anti-A	No	Emergent RBC exchange (≈70% RBC replaced; 7U O RBC); steroids; switch to O RBC	Clinical improvement; later donor-origin thyroid autoimmunity treated with replacement	Yes
Reed et al. (2003) (61)	1	A+	O+	anti-A1	Yes	Supportive; dialysis; G-CSF; transfusion	Death on Day 122 post-Tx	Yes
Squires et al. (2014) (58)	1	O+	A-	anti-D	Yes	O RhD− RBC (12U Day +8–+15); supportive	Self-limited; no further transfusion need after +15	Yes
Teshigawara-Tanabe et al. (2021) (60)	2	O+/B+	O+/A+	anti-A anti-B	Yes/No	Prednisolone (↑to 1 mg/kg in Case2) + supportive transfusion; tacrolimus held in Case2; monitoring anti-ABO	Self-limited with blood type conversion: Case1 A→O; Case2 B→A (hemolysis resolved as recipient-type RBC cleared)	Yes
Zaimoku et al. (2013) (55)	5	A+/B+	O+/A+	anti-A anti-B	Yes/No	Supportive; donor-type RBC; immunosuppression per GVHD	1-year OS 20% in PLS vs 75% without; strong association of early IgM with ensuing aGVHD	Yes

The onset of PLS after intestinal transplantation has been reported between day 4 and day 15 post-transplant, with most cases occurring during the first 1–2 weeks and often around the time of engraftment or early graft function (64–67). Hemolysis is typically abrupt and can be severe, presenting with anemia, jaundice, hyperbilirubinemia, elevated LDH, and reticulocytosis. Some patients required multiple transfusions and intensive support; in severe cases, complications such as renal impairment or sepsis have been documented (24). However, most cases eventually resolved spontaneously. The diagnosis is based on the presence of hemolysis in temporal association with donor-derived antibodies. A positive DAT and identification of alloantibodies in serum or eluates are typical, although false-negative DAT results may occur in the context of brisk hemolysis (23, 24). Exclusion of alternative etiologies such as surgical bleeding, infection, thrombotic microangiopathy, or drug-induced hemolysis is essential (67). In the Cambridge cohort, all nine cases were successfully managed conservatively with transfusion alone, and no deaths were attributed directly to PLS (64).

### 3.7 Heart transplantation

Reports of PLS after heart transplantation are exceedingly rare. Our review identified only a single case described in a cardiothoracic transplant unit, in the context of a broader series of cardiothoracic transplants (52). The case involved a graft from a group O donor transplanted into a group A recipient, with hemolysis attributed to anti-A antibodies produced by donor-derived lymphocytes. The patient developed anemia approximately 18 days post-transplant, with a positive DAT and evidence of hemolysis requiring transfusion support. Diagnosis was based on the temporal association of anemia with hemolysis markers and a newly positive DAT, consistent with passenger lymphocyte–mediated hemolysis. The episode was managed with supportive transfusion, and the patient recovered spontaneously. As with other solid organs, PLS after heart transplantation is generally self-limited once donor lymphocytes are cleared.

### 3.8 Mechanism

**Figure 2:**
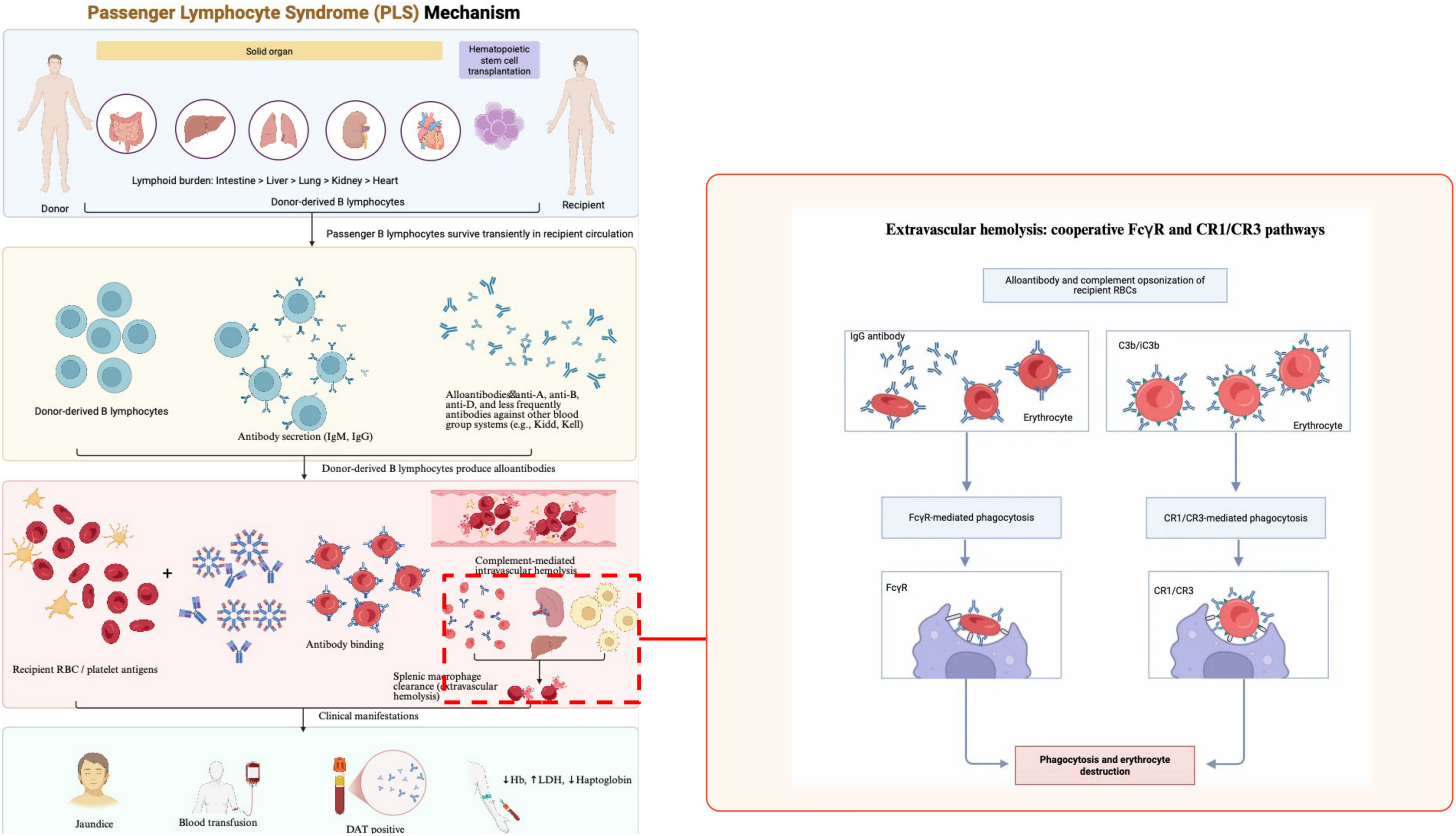
Proposed immunohematologic mechanism of PLS, illustrating donor-derived B lymphocyte migration, antibody production, and subsequent red blood cell destruction through extravascular and intravascular hemolysis.

PLS is initiated by the transfer of viable donor-derived B lymphocytes with the graft (**Figure 2**). These cells can survive transiently in the recipient and are capable of producing alloantibodies directed against recipient RBC antigens (43, 68, 69). Most cases involve antibodies against the ABO system, particularly anti-A, as well as anti-B when group O donors are transplanted into non-O recipients. Less frequently, antibodies against other blood group antigens, such as Rh, Kidd, Kell, and Duffy, and even human platelet or HLA antigens, have been reported (10, 25, 43, 51, 70). Once secreted, these antibodies bind to recipient RBCs or platelets leading to their destruction through two principal mechanisms: (i) extravascular clearance by splenic and hepatic macrophages, mediated by Fcγ receptors recognizing IgG-opsonized cells and by complement receptors CR1 and CR3 recognizing C3b/iC3b-opsonized cells, and (ii) intravascular hemolysis mediated by full complement activation and membrane attack complex formation. In the extravascular pathway, IgG bound to recipient RBCs activates the classical complement cascade, resulting in C3b deposition and its subsequent cleavage to iC3b on the red cell surface. These complement fragments are recognized by CR1 and CR3 (CD11b/CD18) on hepatic Kupffer cells and splenic macrophages, facilitating phagocytosis independently of or in cooperation with FcγR-mediated clearance. Both pathways may operate concurrently, with the relative contribution of each depending on antibody class, subclass, titer, and complement-fixing capacity (19, 26, 43, 49). Not all donor-derived antibodies are clinically pathogenic; low-titer or low-affinity antibodies may yield DAT positivity without hemolysis, highlighting the spectrum from subclinical serologic findings to overt clinical PLS.

Donor sensitization through prior transfusions or pregnancies has been identified as a risk factor for PLS, likely reflecting the transfer of primed donor B cells, presumed to include memory subsets, although direct cellular evidence is lacking (43, 68). Alloantibodies usually appear within 5–15 days post-transplant, which is consistent with a secondary immune response mediated by donor memory B cells; however, rare delayed cases have been described up to 3–4 months after transplantation (20, 23, 68, 69). The antibodies involved are predominantly of the IgG class, while IgM and complement deposition (e.g., C3d positivity) have also been documented in severe cases (29, 38, 60).

Microchimerism studies support the concept that donor-derived B cells can persist for weeks to months in recipient lymphoid organs, thereby sustaining antibody production beyond the immediate postoperative period (32). Notably, donor-derived lymphocytes may exit the graft within minutes to hours following transplantation and preferentially home to recipient lymphoid organs such as the spleen, lymph nodes, thymus, and bone marrow. Under immunosuppressive therapy, donor marrow-derived cells may reside in these compartments for approximately 2 weeks before redistribution, which may explain why studies limited to peripheral blood sometimes underestimate their persistence (34). Finally, calcineurin inhibitor–based immunosuppression primarily targets T-cell function and may not fully suppress activated donor B cells, which explains why PLS can occur despite adequate prophylaxis (44).

The risk and phenotype of PLS vary substantially according to the transplanted organ, reflecting differences in several key immunobiological variables. First, the amount of donor lymphoid tissue transferred with the graft is a primary determinant of PLS risk. The intestine contains the largest mass of gut-associated lymphoid tissue (GALT), including Peyer’s patches and mesenteric lymph nodes, followed by the lung with its bronchus-associated lymphoid tissue (BALT), and the liver with its large population of intrahepatic lymphocytes and Kupffer cells. In contrast, kidney grafts contain comparatively few lymphocytes, which accounts for the lower PLS incidence of approximately 9% in renal transplantation (22, 66, 69). Second, the donor memory B-cell burden influences the magnitude and rapidity of alloantibody production. Donors previously sensitized through transfusion or pregnancy are more likely to harbor primed memory B cells capable of mounting a rapid secondary immune response upon transfer to the recipient. Third, organ-specific immunologic features further modulate PLS expression. In liver transplantation, soluble ABH antigens related to the donor’s secretor status may partially neutralize circulating antibodies and attenuate hemolytic severity. The liver’s unique tolerogenic microenvironment, including hepatic stellate cells and regulatory immune populations, may also influence donor lymphocyte behavior. Fourth, immunosuppressive regimens vary across transplant types and differentially affect donor B-cell activity. Calcineurin inhibitors primarily target T-cell function and may inadequately suppress activated donor B cells, whereas regimens incorporating anti-CD20 therapy (rituximab) or alemtuzumab induction may more effectively deplete passenger lymphocytes. Cyclosporine-based regimens appear more permissive for B-cell activation compared with tacrolimus, which may partly explain differences in PLS incidence across centers and eras (15, 46). In lung transplantation, Kohl et al. demonstrated that PLS-mediating antibodies included anti-A_1_, anti-A, anti-A, B, and anti-B, with the rare anti-A, B specificity confirmed through eluate reactivity against B red blood cells in group A recipients. Plasma IAT alone frequently suggested anti-A_1_ as the sole specificity, underscoring the importance of eluate testing for complete antibody characterization. Additionally, rarer non-ABO specificities including anti-HLA and anti-Kell have been described (53). In kidney transplantation, PLS is less frequent but may present atypically, such as with gastrointestinal ischemia or Coombs’-negative hemolysis (49). In HSCT, donor B cells can ultimately reconstitute the immune system, resulting in prolonged or recurrent antibody production and pathophysiology overlapping with graft-versus-host disease (26). Therefore, the clinical expression of PLS is determined not only by graft lymphoid content but also by organ-specific immunologic features. Beyond RBC antigens, donor-derived antibodies against platelets and HLA have been implicated in post-transplant thrombocytopenia and alloimmune complications (10). Collectively, PLS can be considered a transient, donor-derived graft-versus-host–like reaction that primarily manifests as hemolysis but can also target other hematologic lineages. Although classically directed against RBC antigens, donor-derived antibodies may also target platelet antigens, leading to post-transplant thrombocytopenia, or HLA molecules, particularly after lung transplantation, thereby expanding the pathophysiologic spectrum of PLS beyond hemolysis (10, 53).

Notably, laboratory markers of hemolysis such as elevated LDH and bilirubin and decreased haptoglobin are not specific for PLS and may be confounded by postoperative conditions including hematoma resolution, surgical bleeding, and hepatotoxicity, underscoring the need for serologic confirmation through DAT and antibody identification.

### 3.9 Proposed diagnostic workflow

Based on the diagnostic approaches described in the included studies, we propose the following stepwise workflow for the identification of PLS in recipients of minor blood group-incompatible grafts.

Step 1: Identify at-risk patients. All recipients of minor ABO- or non-ABO-incompatible transplants should be flagged at the time of transplantation, particularly those receiving grafts from group O donors.

Step 2: Monitor for hemolysis during the high-risk window. Hemoglobin, LDH, total bilirubin, and haptoglobin should be monitored at least every 2–3 days during the first 3 weeks post-transplant. An unexplained hemoglobin decline of more than 1–2 g/dL, rising LDH, or falling haptoglobin should prompt further investigation (22).

Step 3: Perform DAT. A positive DAT (IgG and/or C3d) in the appropriate clinical context is the serologic cornerstone of PLS diagnosis (18, 29).

Step 4: Identify donor-derived antibodies. Antibody screening and identification should be performed on patient plasma and, when available, on red cell eluates. Eluate testing is essential to detect antibodies that may not be apparent in plasma, such as anti-A or anti-A, B masked by the higher antigen density of A_1_ test cells (22, 35).

Step 5: Exclude alternative etiologies. Other causes of post-transplant hemolysis must be systematically excluded, including thrombotic microangiopathy, surgical bleeding, infection-related hemolysis, drug-induced cytopenia, and acute rejection. Notably, laboratory hemolysis markers may be confounded by concurrent postoperative conditions such as hematoma resolution (70).

Step 6: Classify PLS severity and initiate management. Based on the findings from Steps 2–5, classify PLS as mild, moderate, or severe and initiate the corresponding management approach as outlined below. This proposed workflow is synthesized from the diagnostic approaches described across the included studies and is intended as a practical guide rather than a validated clinical guideline. Prospective evaluation in multicenter settings is warranted to determine its diagnostic performance and clinical utility.

### 3.10 Proposed severity-based management approach

Based on the cases reviewed, we propose a stratified management framework for PLS according to clinical severity. Mild PLS is characterized by a modest decline in hemoglobin (1–2 g/dL) with positive DAT and detectable donor-derived antibodies but without symptomatic anemia or transfusion requirement. Management is expectant, consisting of close monitoring of hemoglobin, hemolysis markers, and serial serologic testing. Transfusion with donor-compatible (antigen-negative) RBCs should be available but may not be necessary. Most mild cases resolve spontaneously within weeks as donor lymphocytes are cleared (29). Moderate PLS is defined by a hemoglobin decline exceeding 2 g/dL or symptomatic anemia requiring transfusion support. The cornerstone of management is transfusion with donor-type or antigen-negative RBCs, as recipient-type units may exacerbate hemolysis (29). Serologic crossmatching should guide RBC unit selection. Corticosteroid escalation may be considered, particularly in cases involving Rh antibodies, although evidence supporting efficacy remains limited (49, 72).

Severe PLS encompasses life-threatening hemolysis with hemodynamic instability, acute kidney injury, or multiorgan dysfunction. In addition to antigen-negative RBC transfusion, escalation to immunomodulatory therapy is warranted. Reported interventions include plasmapheresis or red cell exchange to rapidly reduce circulating alloantibody burden, intravenous immunoglobulin (IVIG) to modulate Fc receptor–mediated clearance, and rituximab to deplete donor-derived B cells producing the pathogenic antibodies (21, 46, 66, 69). In refractory thrombocytopenic variants, efgartigimod has been used successfully (38). The choice among these interventions should be guided by the predominant immunopathologic mechanism: complement-fixing antibodies causing intravascular hemolysis may respond better to plasmapheresis, whereas FcγR-mediated extravascular clearance may be modulated by IVIG or corticosteroids. Across all severity grades, awareness that calcineurin inhibitor–based immunosuppression primarily targets T cells and may inadequately suppress donor B-cell activity should inform clinical decision-making. Prophylactic rituximab administration before ABO-mismatched transplantation has shown promise in eliminating PLS in some cohorts, although breakthrough cases have been reported (83).

### 3.11 Limitations

Several limitations of this review should be acknowledged. The majority of included studies were single case reports or small case series, which inherently introduces publication and severity bias. Severe or unusual PLS presentations are more likely to be published, whereas mild or self-limiting episodes that resolve with supportive care alone are underrepresented in the literature. Consequently, the clinical spectrum described in this review likely overestimates the severity of PLS, and the reported frequencies of complications such as renal failure or graft loss should be interpreted with caution. Furthermore, true incidence estimates remain unreliable, as only a few cohort studies with systematic screening have been conducted. The prospective data from Kohl et al. illustrate this point: active immunohematologic surveillance detected PLS in 30.77% of at-risk lung transplant recipients, compared with 18.18% when testing was performed only on clinical indication. Multicenter prospective studies with standardized diagnostic protocols are needed to define the true incidence and severity distribution of PLS across transplant settings.

## 4 Conclusion

PLS is a rare but clinically significant complication across both solid organ and hematopoietic stem cell transplantation. It is most often mediated by ABO antibodies, arises within the first 1–3 weeks after transplantation, and is usually self-limited as donor lymphocytes are cleared. However, severe or even fatal cases occur, underscoring the importance of early recognition and timely management. Awareness of its incidence patterns, antibody spectrum, and laboratory features across different organ types can aid clinicians in distinguishing PLS from other causes of post-transplant anemia. Greater vigilance, systematic reporting, and collaborative studies are also needed to refine prevention, improve therapeutic strategies, and optimize patient outcomes. Given that PLS is likely underdiagnosed, systematic serologic surveillance including DAT and antibody screening during the first 2–3 weeks post-transplant is recommended for all recipients of minor ABO-incompatible grafts. Notably, laboratory hemolysis markers such as elevated LDH and bilirubin may be confounded by postoperative conditions including hematoma resolution, and therefore serologic evidence of donor-derived antibodies should be considered the diagnostic cornerstone. Future directions include establishing standardized diagnostic criteria, identifying biomarkers predictive of clinically significant hemolysis, and evaluating prophylactic or preemptive B-cell–directed therapies in high-risk settings.
